# Report of the Committee on Practical Medicine

**Published:** 1858-10

**Authors:** F. K. Bailey

**Affiliations:** Chairman


### ARTICLE VIII.

### REPORT OF THE COMMITTEE ON PRACTICAL MEDICINE.

By F. K. BAILEY, M. D., Chairman.

The constitution of the Illinois State Medical Society declares that the Committee on Practical Medicine "Shall prepare an annual report on the more important improvements effected in this State in the management of individual diseases; and on the progress of epidemics; referring, as occasion requires, to medical topography, and to the character of prevailing diseases in special localities during the term of their service."

Soon after the meeting in June last, circulars were sent to all the medical men in the State, whose post-office address could be ascertained, and the same circular was published in the *N. W. Medical and Surgical Journal.* The presumption is, that a majority of the practitioners in the State who might be expected to respond, are aware of the existence of a Committee on Practical Medicine. Some, as will be perceived, have favored us with communications, but comparatively few have have seen fit to respond.

For such as have been furnished, the Committee would express their gratitude, not only on their own account, but also of the profession generally. Besides such extracts thus furnished, we shall embody some extracts from medical journals, and remarks made by your chairman.

The duty of strict observation on the part of the practitioner of medicine, is of undeniable importance. A close scrutiny at the bed-side of the sick is indispensable, both to the success and satisfaction of the physician, and the ultimate welfare of the patient.

Medical practice is in the hands of thousands of men scattered through our cities and country towns, who, in the discharge of their daily duties, are conversant with the different forms of diseased action.

The course of treatment adopted in the case of those who recover, is seldom made public; nor is the world any wiser, as to the reason why in fatal cases, the result was not otherwise.

Like the camel in the desert, who is constantly making footprints in his sandy path, only to be obliterated by the next breeze, so, with the majority of practitioners, they from day to day are travelling in a track which is never afterwards seen.

With our neighbors across the way in the legal profession, it is otherwise. Their acts and decisions, although of much less importance as far as the good of the human race is concerned, are carefully noted, and preserved for future reference and precedent.

With our profession, we are pained to say, much is lost, which, if made a matter of record, might in similar cases be safely appropriated; and even the errors that are daily committed, if noted, might serve as warnings to others, like the lone rock in the ocean path, which, although the occasion of now and then a shipwreck, serves as its own signal of danger to those who are on the look-out. Much that is written upon medical subjects, is from the pens of men residing in the large cities. Cases occurring in hospitals and similar institutions are carefully noted, and also those occurring in private practice. A great variety of cases is met with in country practice, and equally interesting to the profession.

But the practitioner, from a feeling of timidity, may hesitate to give the symptoms and treatment of an interesting and important case, although fully competent to do so. He may have found "a more excellent way" of treating a disease, which, if made public, would be gladly followed by others. This modesty, if indeed it deserves so mild a name, is not commendable, for an idea may be suggested to the profession by some one practising in a rural district upon our vast prairies, which, if made known, might be hailed with great interest by all practising the healing art. A fact brought to light in treating the child of some family residing in our own State, may be the occasion of bringing joy to the hearts of some royal family in the Old World. A truth is the same whether brought out by one practising in our midst, or at the head of some European hospital, or popular medical school. A paroxysm of intermittent may be the same, whether occurring on the banks of the Illinois or Seine. The phenomena seen and felt in a severe form of neuralgia may not differ, whether met with in some Western village, or in London.

There are doubtless many men in our own State, who, if called upon to prescribe for a patient in the city of Paris, might even astonish the wise men of France, by their display of good sense.

These thoughts are thus expressed with the hope of stimulating our brethren to the importance, not only of observing closely, but of communicating to others, the result of their observations.

We heartily refer our friends to some very excellent remarks upon the topic last spoken of, by one of the editors of the *Chicago Medical Journal*, in the April number. It must be admitted that the profession of our State are becoming awake to this important subject, and we find that medical societies, both county and district, are being organized, the members of which are contributing something to our medical literature.

In reply to the interrogatories in our circular, Dr. F. R. Payne, of Marshall, writes:

"During the last summer and fall, we had more than the usual amount of rain, but it fell in gentle and often-repeated showers, not sufficient to thoroughly saturate the ground and fill up the ponds; the consequence was, that we had but few malarious diseases. In the latter part of the fall, and during the winter months, typhoid fever was a common disease. Not typhoid grades of remittent fever, and pneumonia, but typhoid fever in all its purity. I have treated fevers in this locality for sixteen years, and feel thoroughly satisfied that my diagnosis is correct. That this fever is to a certain extent superseding endemic fevers, I have no doubt; and I also firmly believe that out of the river and other low bottoms, and marshy lands, it will, in a few years, entirely take the place of remittent fever.

"I will not enumerate all of the symptoms of this disease, but present those that occur early, and constitute my principal means of forming a correct diagnosis. When the typhoid element (whatever it may be) begins to exert its morbid effects upon the system, the patient complains of general debility, has no inclination to active mental or physical exertion. When he rests upon his bed for a few hours, he begins to doubt the existence of disease; but an attempt to move about, will again arouse his fears. The skin is dry, particularly in the palms of the hands and the soles of the feet; pulse soft, and slightly accelerated. When the head is raised, and the arm rests upon the elbow, we discover a tremulous motion; tongue coated, and dryness about the lips; a gurgling sound usually in the right iliac fossa: this symptom occurs early in the disease in about two-thirds of the cases. In from four to ten days, these symptoms increase in severity; pulse from 85 to 120; secretions, except from the ilium, diminished; the white coat upon the tongue turns dark and dry; the edges are red; intellect dull and confused; by close examination we frequently find streaks of bloody mucus in the stools, and nearly always if active cathartics have been administered. The pulse remains nearly the same, seldom varying two beats in the twenty-four hours. These symptoms are followed by the more alarming characteristic symptoms of typhoid, as laid down in the books, and if not properly treated, usually terminate in death in from sixteen to forty days.

"As to the cause of this affection, we will not venture an opinion. It is very clear, that let the cause be what it may, the nervous system is made to suffer in a powerful manner from its effects. It cannot be a specific poison, but we are inclined to the belief that the cause may be either general or local.

"Within the last year we have adopted a course of treatment of our own, in some very important respects, with the most satisfactory results: but few cases have died, in fact none where the treatment was fully carried out from the inception of the disease.

"We commence the treatment with a moderate dose of blue mass, and if there is much diarrhea, we combine opium with it. If this does not operate in six or eight hours, give oil and turpentine. Sponge the surface with warm alkaline water. After the operation of the cathartic, we give ipecac. from 3 to 6 grs., opium from 1 to 3 grs., every three hours. If the pulse is over 100 per minute, alternate the powders with from 5 to 7 drops tr. veratrum viride; but if the pulse is under 100, and the tongue dry, we substitute the turpentine emulsion for the veratrum viride. By this means we, if possible, prevent an operation on the bowels for from three to six days.

"During this time, we proscribe all solid articles of diet; boiled milk thickened with flour is a favorable article of diet. If the bowels become somewhat disturbed, with no action in them to six days, we give oil and turpentine, and omit the powders, but resume them immediately after the oil operates.

"To promote the secretion from the kidneys, infusion of *uva*

*ursi* is the best preparation. If profuse hemorrhage comes on, we add to our powders acet. plumbi. Strong tr. valerian is the best remedy for *subsultus*. Quinine in this continued form of fever is positively injurious; it increases the subsultus and delirium, and in no case do we believe will it interrupt the fever. We remove from the room all causes of excitement, and prohibit visitors from entering the sick chamber. If the symptoms are not urgent, we give no medicine from 10 o'clock p.m. to 5 a.m., and direct the nurse to lie down near the patient with all the lights extinguished. These directions we believe are of great importance.

"It may be well to remark that Marshall is situated on the dividing ridge between Big and Mill Creeks, about two miles distant from either stream. The land is rolling, and usually dry and compact. The soil is dusky white, and deficient in humus, consequently does not contain an atmosphere of carbonic acid, a fact which it might be well to note, in looking for the proximate cause of typhoid fever.

"This winter we have had quite a number of cases of pneumonia, but the disease has readily yielded under our treatment; and as it is the first winter we have used the *veratrum viride* in this affection, we will briefly state our manner of using it, and record our experience thus far, in favor of the remedy. We, in an ordinary case of pneumonia, at our first visit, make the following prescription:

Hyd. sub. mur., . . grs. xx.
Pulv. Doveri, . . . " x.

M. F. pulv. ij., to be given three hours apart.

"In an hour and a half after the first powder is taken, give five or six drops veratrum, and continue every three hours. In six hours after the last powder is given, if there is no operation from the bowels, give castor oil.

"When this operates, alternate the veratrum with pulv. Doveri 3 or 4 grains, and opium one grain, with a large cataplasm over the chest.

"Under this treatment usually (not in every case) in twenty-four or thirty-six hours, the pulse is brought to the normal standard; the patient is bathed in a profuse perspiration, and the disease terminates speedily in resolution. When there is a typhoid element at work in the system, the pulse will come down, and the violence of the disease is greatly mitigated, but convalescence is not established until from nine to sixteen days. Suffice it to say, that we have not lost a case, with or without complication, since the adoption of this plan of treatment, and we have had twenty cases, when the diagnosis was positive. In ordinary cases, when called early in the disease, we seldom find it necessary to repeat the sub-muriate. In case the bowels are not sufficiently lax, we use every twenty-four hours some mild cathartic.

"The above plan meets the indications in the treatment of this disease better than any we have hitherto adopted. Some cases may require more than six drops of veratrum, but we have never found it necessary to give over eight drops. We have not used calomel with as much liberality as we did a few years ago, from the fact that patients seem more susceptible to its constitutional effects; and farther, we do not belong to that class of physicians who believe that two diseases cannot exist in the system at the same time."

Dr. D. W. Stormont, of Grand View, Edgar County, and one of the Committee on Practical Medicine, writes respecting the topography of his district:

"This is a pleasant village, of about five hundred inhabitants, situated in the southwestern part of Edgar County, and in the southern edge of a portion of the Grand Prairie, which here has a general direction of N.E. and S.W.

"The prairie adjacent to the timber is in a high state of cultivation; and the surface is gently undulating, with originally but few sloughs or ponds, and these are now well drained. Farther out, say from three to ten miles, the surface, though not flat, is generally more level; consequently more ponds and sloughs are found; and the improvements being of more recent date, and the cultivation more imperfect, the water collects in the spring, and in many places stands throughout the summer. The soil is a black loam, which varies in depth from eighteen inches to several feet.

"To the S. S.E. and S.W. he have a heavy body of timber, consisting principally of oak, hickory, maple, walnut and poplar.

"The surface, compared with the prairie, is broken, even hilly in many places. There are numerous small streams or creeks, which rise in or near the prairie, and running a south-easterly direction, empty into the Wabash, at distances varying from twenty-five to seventy-five miles. The soil is thinner than on the prairie, but productive. The bluffs bordering on the creek bottoms, present limestone in considerable quantities; occasionally an inferior quality of coal is found. Several springs throughout the timber are strongly chalybeate.

"This community is almost exclusively an agricultural one; grain and stock raising the principal business. For intelligence, industry, morality, and consequently thrift, we think it will compare favorably with any other in the State.

"From this imperfect sketch of the topography of this locality, you will see we are rather favorably situated for observing and comparing the different forms of disease, as they occur in highly malarious, or in the more salubrious districts; and for noting the different phases the same disease may assume in these respective localities."

Dr. S. has above given a description of the varieties of the surface, viz.: that lying in, and adjacent to, the timber belts, which so gracefully diversify the face of our beautiful State, and that of the prairie at a distance from the groves, it is at present, and is becoming more so every year, an interesting inquiry, as to which is the most healthy part of the country, the groves or the prairie. It is well known that the first settlers of this State selected a residence either in or near a grove of timber, on account of having fencing and fuel at hand, and also because water could be found more conveniently. Recently, however, many are settling upon the broad prairie miles from a tree or stream.

The comparative healthiness of the different localities might be easily ascertained; and perhaps facts showing that diseases prevail in each, differing not only in type, but also in kind, might be presented.

Dr. S. further states that "The winter of '56 and '57 was long and cold. The spring following was cold, wet and backward. The summer and fall were also cool, with frequent showers of rain. November was cold, with considerable snow. December '57 and January '58 were mild and pleasant, with but little rain or snow. February '58 was cold, with abundance of snow. March was warm and dry, and the healthfulness of this region has been much above the average."

*Periodical Fevers.*—"These are our common endemics of the summer and fall months, occurring most frequently, as would be expected, on the prairie. Last season there were fewer cases, but they were generally more obstinate than usual. The intermittents presented a large proportion of congestive or malignant cases, or of cases threatening that form, requiring a prompt and heroic treatment; but differing in no wise from that usually adopted under such circumstances, unless it was a longer continuance in the use of quinine.

"The remittents were obstinate, frequently assuming a low grade, with indistinct remissions, torpid liver, and indeed a locking up of the liver generally;—sometimes accompanied with prominent typhoid symptoms, as cephalalgia, slight delirium, subsultus, parched tongue, and perhaps meteorism, but seldom diarrhea. This condition of things did not obtain in all cases, but in those only which were not seen in the commencement, or were not then treated with *promptness* and *energy*.

"Under the use of alterative doses of calomel, with diaphoretics,—Dover's powders or spirits nit. dulc.,—and quinine in full doses, the symptoms would gradually yield, and in ten or fifteen days convalescence would be fully established. Of course other means, such as cold or tepid sponging, were employed in every case, cupping or blistering when indicated, etc.; but small doses of calomel, not carried to ptyalism, however, with quinine and Dover powders, each grs. iv. or. v. every three or four hours, with spirits of nitre, occasionally, was the general treatment.

"This form of intermittent is not encountered every year, and is generally confined to the prairie. It is popularly called typhoid fever; but is not regarded by the profession, so far as I can learn, as the 'Typhoid' of Louis, or 'Enteric' of Wood; though in its latter stages it is difficult to distinguish from it. In these cases the commencement of the attack is more abrupt, the biliary derangement, and the periodicity more apparent, while the cephalic, nervous and abdominal symptoms are less apparent, and the disease is altogether less protracted, and more under the control of remedies, than in the genuine or specific typhoid.

"Typhoid or enteric fever prevailed to some extent during the fall and early part of the winter. Of late years, it is often encountered in the timbered region, generally in the winter months, presenting the aggregate symptoms detailed by Bartlett and Wood. But I am not confident that I have seen it in its specific form originate on the prairie until last fall.

"There was one peculiarity observed in these cases on the prairie, which I have not found in those occurring in the less malarious districts in the timber, that was a periodical element often quite marked. It was generally quotidian in type, but sometimes more apparent on alternate days, and sometimes so prominent as to obscure the diagnosis, rendering it doubtful whether it was the true enteric, with a malarious disease superadded, or the low grade of remittent mentioned above.

"Here the effect of remedies was a valuable diagnostic. If it was a simple remittent, the quinine treatment before alluded to would soon ameliorate all the symptoms, the pulse becoming more full, softer and slower, the secretions gradually established, and convalescence soon apparent. On the other hand, if the case should be one of mixed disease, these doses of quinine will break up its periodical element, and leave the enteric fever to run its course without this dangerous complication, but uncontrolled by this remedy. Ordinarily, after the malarious disease is arrested, those anti-periodic doses are illy borne, producing irritability of pulse, and aggravating the cephalic, nervous and gastric symptoms.

"A residence in a malarious district creates a tolerance, even a demand for quinine, in very many cases of every class of disease, at all seasons of the year. And a proper appreciation of this fact is of vital importance to successful practice in such regions."

Dr. S. has intimated above that the timbered regions are less malarious than the prairie. Allow us to remark that facts coming under our notice go to show that the prairie is the most free from our usual malarious diseases. We find in the "timber" not only a much greater amount of vegetable matter, such as leaves, rank weeds and branches of trees, but also a stream of water, the banks of which are at times overflowed, leaving a quantity of water to become stagnant. The wind being less strong in the timbered regions, it follows that the heat of the sun will be greater, and consequently decomposition of vegetable matter more rapid.

On the open prairie there is a constant breeze more or less strong, which, as a matter of course, must tend to dissipate miasm. It is a fact, too, according to our observation, that families who once lived in the groves, have, on removing to the more airy position upon the prairie, suffered much less from sickness. This is also the opinion of intelligent physicians with whom we have conversed, and who have had good opportunities for observation.

"The peculiar eruptions—rose-colored spots—was observed in about one-third of my cases, though they were not searched for carefully.

"On the treatment I have but little to say. In my experience the expectant method has in general been the most satisfactory. But I almost uniformly administer one or two grains of quinine with one-fourth grain each of opium and ipecac., three times a day, commencing early in the attack. It is generally well tolerated, and gives tone to the digestive organs. I have no cause for gratulation in the use of alcoholic stimulants. Blisters I have not found as troublesome as the authorities would make us believe. But I am careful to have them taken off as soon as vesication has commenced, and the process completed by means of a poultice. I have seen signal advantage follow their use, both as revulsives and stimulants. Mercury I administer to some extent in every case, generally in form of blue pill, but never designedly to ptyalism.

"Pneumonia is common in the winter and spring, and occasional cases are encountered at all seasons of the year. In the open sthenic variety, we find the usual symptoms of chill, succeeded by headache, high fever; full, frequent pulse; hurried respiration; pain in the breast or side of varying intensity; cough more or less severe; the expectoration viscid, and perhaps bloody or rusty colored; crepitation, followed by bronchial respiration and dullness on percussion. In these cases, as a rule, I do not employ general bloodletting; but after the operation of a mercurial cathartic, the patient is put upon pulv. opii, 1 to ij. grs., tart. ant. and potass, $\frac{1}{6}$ gr., to be given every two or three hours until rest is obtained, when the interval may be prolonged to four or five hours. The rule in administering the opium is to vary its amount and frequency by the severity of the pain or local disease; and after that has been relieved, to continue it at such intervals as may be necessary to keep up an opiate influence. The local abstraction of blood by cups, and the application of poultices to the chest, are often attended with marked benefit. After the intensity of the symptoms has been somewhat moderated, a large blister may be applied over the diseased lung; but this is not always required when the opiate treatment has been commenced early. A few grains of calomel, from six to eight, are administered every day or every other day, with an opium powder at bedtime, and, of course, purgations when demanded. Under this general treatment, uncomplicated sthenic pneumonia, as encountered here, is rarely if ever a fatal disease.

"Typhoid or asthenic pneumonia never prevailed to any extent in this immediate vicinity until the spring of 1856, although it may, perhaps, have existed previously in adjacent neighborhoods.

"During the summer and fall of 1855, nearly every person in all this region of country was attacked with intermittent or remittent fever. The following winter was uniformly very cold until about the 1st of March, when warm weather set in rather abruptly.

"At this time typhoid pneumonia began to appear, and cases continued to occur for several weeks; many died. It was most frequently found on the prairie, where occurred the worst cases. During the summer and fall of 1856 there was again considerable malarious disease. The winter following was also very cold; and in the spring of 1857 we again had typhoid pneumonia, but not so extensively nor so fatally as in the previous spring. The past year was comparatively free from periodical fevers; the winter just closed was mild and open, and I have not heard of more than three cases of typhoid pneumonia in the neighborhood.

"The symptoms of this were somewhat different from those of the former variety of pneumonia. It usually occurred in those whose general health had been impaired, or constitutions were debilitated by previous disease. In every case so far as my observation extended, there was enlarged spleen readily detected by palpation or percussion.

"Compared with the sthenic variety, the attack was more insidious, the patient complaining for two or three days of general malaise and anorexia; the chill was of longer duration, and the reaction more imperfect, the face and trunk often being hot, even imparting a pungency to the touch, while the extremities were rather cool. Severe headache, frequently attended with a dullness, or with slight delirium, from the onset; restlessness; pain in the back, with general muscular soreness and debility; the pain in the side dull, and sometimes wanting; tongue red on the tip and edges, otherwise coated with a whitish or yellowish fur, which soon assumed a dark color, dry and cracked; sordes would collect on the teeth and gums; in many cases there was slight hemorrhage from the nose; pulse weak and frequent; respiration hurried and irregular; frequent sighing; cough short and generally loose; expectoration less viscid, sometimes dark, frequently thin and watery or bloody, often almost pure blood; crepitation indistinct and soon supplanted by mucous rale, and dullness on percussion. Commonly the right lung was the principal seat of the disease; but in every instance, I believe, both were involved.

" *Treatment.*—Generally the patient was put from the first upon the following:

| | | |
|---|---|---|
| ℞ Sul. Quinia, | . . . | grs. xvj. to xx. |
| Calomel, | . . . . | " xv. |
| Pulv. opii, | . . . | " ij. |
| Ipecac., | . . . . | " j. |

"Divide into four powders, and give one every three hours. Sometime even, this amount of opium could not be borne, but when there was much pain, and little or no stupor, it could be increased to advantage. If there was much debility, camphor was added. When the tongue began to clean and moisten, or there was danger of ptyalism, the mercurial was withdrawn; but the quinia was continued till the last. If the bowels were not freely open daily, purgatives were administered; generally castor oil and spts. turpentine. It is important not to suffer the patient to lie too long in one position, which he will do if undisturbed. He should be frequently turned from the back to the side, etc., and supported in different positions by pillows. Dry cupping, sinapisms or hot poultices to the chest, and hot pediluviæ or mustard to the extremities, were sometimes used with benefit. But large and repeated blistering, generally from the commencement, was employed with most signal advantage. A blister, 8 by 10 inches, or larger, was applied over the seat of the disease, and suffered to draw well. After the excitement thus induced had somewhat subsided, if the amendment was not marked, it was applied to the other side. A strong irritation and depletion were kept up by these means (by reapplying the blisters if necessary), until convalescence was established. In the meantime, if the circulation should be flagging, or reaction imperfect, blisters were applied to the ankles and wrists also. This looks like a skinning process; but in my experience, it was second to no other means employed towards a favorable issue.

"Strangury was apt to result from this repeated and excessive blistering, but it was a sure indication of returning health; every case recovering after this effect was produced; the amendment dating from that period.

"I have no data from which to give the ratio of mortality; but under the treatment here detailed, commenced early, and perseveringly used, it was small. The great mortality mentioned,

