
JOURNAL INFORMATION
==============================
NLM Title Abbreviation: Chic Med J
NLM Journal ID: 0027576

The Chicago Medical Journal

Publisher: Biomedical Journal Digitization Project

ARTICLE INFORMATION
==============================
PMCID: PMC9721723
PMID: 37411177
Article version: 1
Subjects: Editorial

Omission

Print publication date: 1858 Oct
Volume: 15
Issue: 10
First page: 544
Last page: 544
License: This work is free of known copyright restrictions. For more information, please see PMC Back Issue Digitization.
License URL: https://creativecommons.org/publicdomain/mark/1.0/

Erratum for: Report of the Committee on Practical Medicine 15 10 10 1858 519 532 The Chicago Medical Journal PMC9721712 37411169

==============================

### PROFESSIONAL APPOINTMENTS.

The chair of surgery in the National Medical College, at Washington, made vacant by the resignation of Prof. May, has been filled by the appointment of Dr. John G. F. Holston. The chair of chemistry in the Virginia Medical College has been filled by the appointment of Dr. James B. McCaw.

### NEW YORK STATE INEBRIATE ASYLUM.

The State of New York enjoys the honor of being the first in our country to appropriate the means for establishing a State institution for the reception and treatment of confirmed *inebriates.* It has been located in the beautiful village of Binghamton, in the southern part of the State, and the corner stone was laid on the 24th of September last. Rev. Dr. Bellows, Dr. John W. Francis, Hon. Edward Everett and Hon. D. S. Dickinson, took part in the exercises.

---

OMISSION.—At the bottom of page 532, the words "to be continued" ought to have been inserted.

### FRESH VACCINE VIRUS,

**Constantly on hand and for Sale, by**

J. H. REED & CO., WHOLESALE DRUGGISTS, 144 & 146 LAKE STREET.

### GALE BROTHERS,

### APOTHECARIES AND CHEMISTS

**202 Randolph Street, Chicago.**

We are prepared to furnish Physicians with Medicines and Chemicals of the most reliable qualities, selected and prepared expressly for our own dispensing department.

New and rare Chemicals and Pharmaceutical Preparations furnished in quantities to suit Practitioners, to whom we recommend an examination of our stock.

### ARTIFICIAL EYES!

We have a large assortment of *Artificial Human Eyes,* of the most approved manufacture.

**GALE BROTHERS,**
APOTHECARIES AND PHARMACEUTISTS,
202 Randolph St., Chicago.

### TO THE MEDICAL PROFESSION.

The subscribers would call the attention of physicians to the annexed list of Fluid Extracts, which we have been induced to prepare, from the difficulty of obtaining such preparations of a reliable character, and to obviate the great inconvenience of being dependent on distant manufacturers for articles of every day use by physicians.
